# Clinical and radiological outcomes following hybrid surgery in the treatment of multi-level cervical spondylosis: over a 2-year follow-up

**DOI:** 10.1186/s13018-015-0330-5

**Published:** 2015-12-16

**Authors:** Ji-Sheng Shi, Bin Lin, Chao Xue, Hai-Shen Zhang, Zhi-Da Chen, Zhong-Sheng Zhao

**Affiliations:** Department of Orthopedics, Hospital of Orthopedics, The 175th Hospital of PLA, Southeast Hospital of Xiamen University, No. 269 Zhanghua Road, Zhangzhou, 363000 Fujian People’s Republic of China

**Keywords:** Anterior cervical discectomy and fusion, Multi-level cervical spondylosis, Hybrid surgery, Cervical disc arthroplasty

## Abstract

**Background:**

The optimal surgical approach for treatment of multi-level cervical disc disease is currently widely debated. Anterior cervical discectomy and fusion (ACDF) combined with cervical disc arthroplasty (CDA) has been presented as a treatment approach, but to date, there are few reports with adequate clinical and radiological data for this hybrid surgical procedure. The goal of this paper is to assess clinical and radiological outcomes in patients with cervical spondylosis in three contiguous segments after treatment with artificial disc replacement combined with fusion.

**Materials and methods:**

We performed a retrospective review of 36 patients (mean age of 48.6 years) with contiguous three-level cervical spondylosis who were treated with ACDF coupled with CDA (hybrid surgery) between October 2008 and October 2012. Clinical evaluation was based on the Neck Disability Index (NDI), Japanese Orthopaedic Association (JOA) score, and postoperative JOA score improvement rate (IR). Radiographic parameters, angular range of motion (ROM) for C2-C7, and ROM for the superior and inferior adjacent segments were measured before the operation, at 1, 3, 6, and 12 months postoperation, and at the final follow-up evaluation. All cases were followed for at least 28 months (range 28–65 months).

**Results:**

All patients exhibited significant postoperative improvement in NDI and JOA scores compared to preoperative levels (*P* < 0.05), and these improved scores were maintained during the follow-up period. The JOA score improvement rate was 70.83 % at the final follow-up evaluation. The mean C2-C7 ROM of all cases was significantly decreased immediately after operation but recovered to preoperative levels after 12 months (*P* = 0.721). The ROM of the superior and inferior adjacent segments was recovered to preoperative levels after 6 months (*P* > 0.05). One patient required a second surgery for symptomatic adjacent segment degeneration. Neither pseudarthrosis nor other device migration was observed in any patients during the entire follow-up period.

**Conclusions:**

These results indicate that hybrid surgery seems to be a promising, acceptable, and alternative surgical approach for the treatment of multi-level cervical disc disease.

## Introduction

Cervical spondylosis is a common pathological condition that results from cervical spine degeneration and has been shown to cause significant disability and loss of productivity [1]. Research on the surgical treatment of cervical disease has mainly focused on the surgical approach, decompression method, and selection of internal fixation. For the surgical management of cervical degenerative disc disease, spine surgeons have explored anterior surgical approaches, such as anterior cervical discectomy and fusion (ACDF), anterior cervical corpectomy and fusion (ACCF), and cervical disc arthroplasty (CDA), and posterior surgical approaches, such as laminectomy and laminoplasty [2, 3].

Over the past few decades, ACDF has proven to be an effective and acceptable treatment for single- or double-level cervical spondylosis [4]. Nevertheless, ACDF may cause long-term complications, such as activity loss in the surgery segment, which may lead to a higher incidence of adjacent segment degeneration and segmental instability [5]. Furthermore, for patients with multi-level cervical degenerative disc disease, multi-level fusion is more likely to lead to adjacent segmental disease, challenging fusion, and frequent pseudarthrosis [6]. Therefore, CDA was developed to preserve the activity of the surgical segment and to restore the normal biomechanics of the cervical spine [7]. To date, however, there have been few reports on the biomechanical effect of CDA involving three or more levels, and its clinical indications and contraindications are unclear.

Currently, artificial disc replacement combined with fusion (hybrid surgery) has been presented for the treatment of multi-level cervical disease [8]. Unfortunately, there are few clinical data concerning the efficacy of hybrid surgery for the treatment of this disease and the effect of the combined procedure on adjacent segments. There is also a lack of significant data reporting the clinical indications and contraindications of hybrid surgery for multi-level cervical disease, in spite of its significant advantages [9, 10]. We have performed hybrid surgery for the treatment of multi-level cervical disc disease since 2008. This study was conducted to review the clinical efficacy of the surgery and to investigate surgical key points and indications in patients treated with artificial disc replacement combined with fusion.

## Materials and methods

This study protocol was approved by the Research Ethics Board of our institution. Informed consent was obtained from all patients. This study included 36 patients (21 males and 15 females) with age ranging from 39 to 60 years (mean age 48.6 years), who were seen between October 2008 and October 2012 for cervical disc disease involving three contiguous segments. Patients with obvious cervical instability, osteoporosis, significant cervical anatomical deformity, or active infection were excluded. Fusion or CDA was determined preoperatively by anterior-posterior and lateral flexion-extension radiographs, computed tomography (CT) scans, and magnetic resonance imaging (MRI).

**Fig. 1:**
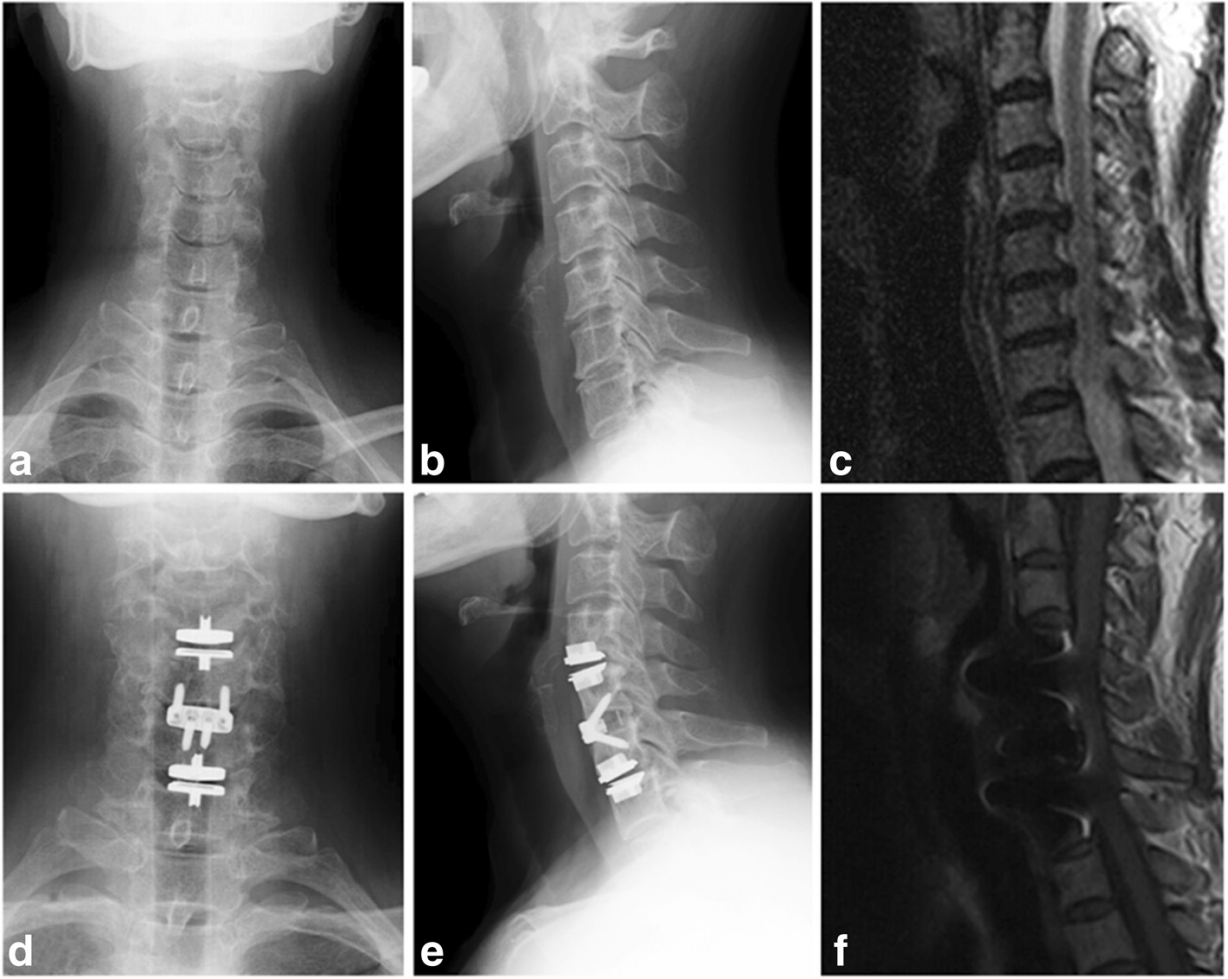
**a** Postoperative anteroposterior and **b** lateral cervical spine radiographs illustrate straight cervical lordosis and vertebral bone hyperplasia. **c** Sagittal T2-weighted MRI demonstrates spondylosis at C4-C5, C5-C6, and C6-C7. **d** Anteroposterior, **e** lateral radiographs and **f** sagittal T1-weighted MRI after the patients underwent cervical disc arthroplasty combined with midlevel anterior cervical discectomy and fusion at 30 months postoperatively

Nine operations were performed with one ProDisc-C disc prosthesis (Synthes Spine, West Chester, PA, USA) and two cages (Stryker, Kalamazoo, MI, USA); 18 operations with two disc prostheses and one ZERO-P (Synthes Spine, West Chester, PA, USA); and nine operations with two disc prostheses and one cage. In one case, the ProDisc-C arthroplasty was performed at C4/C5 and C6/C7, and the ZERO-P was implanted at C5/C6 (Fig. 1).

Data collected included patient demographics and pre- and postoperative information (age, sex, levels, symptoms, follow-up duration, operation time, blood loss, and hospital stay). Clinical evaluation was based on the Neck Disability Index (NDI), Japanese Orthopaedic Association (JOA) score, postoperative improvement rate, radiographic parameters, and angular range of motion (ROM) for C2-C7 and for the superior and inferior adjacent segments. The postoperative improvement rate (IR) was defined as (*b* − *a*)/(17 − *a*) × 100 %, where *a* indicates the preoperative score, *b* indicates the postoperative score, and 17 indicates a normal score [11]. The ROM was measured by the difference in pre- and postoperative Cobb angles as measured at the 1-, 3-, 6-, 12-month, and final follow-up examinations.

### Operative technique

All surgical operations were performed with patients placed in a supine position with the head extended. Controlled general anesthesia was administered, and endotracheal intubation was performed for all patients. First, ACDF was used for the fusion segment through a standard right-sided anterior approach, as described in previous report [12]. Extensive decompression was performed, with removal of the disc tissue, hyperplastic posterior longitudinal ligament, and posterior osteophytes. A cervical interbody fusion cage or ZERO-P was used. Subsequently, replacement segments were operated upon to implant the ProDisc-C artificial disc prosthesis. After the operation, all patients were allowed to wear a neck collar for 4 weeks and to undergo proper functional exercise.

### Statistical analyses

Statistical analyses were conducted using SPSS software, version 19.0 (SPSS Inc, Chicago, IL, USA). Continuous variables are presented as means ± standard deviation (SD). JOA, NDI, and ROM scores were analyzed by Student’s *t* test. Statistical significance was set at *P* value <0.05.

## Results

We operated on a total of 108 levels, including 63 replacement segments and 45 fusion segments. All operations were successful. All cases were followed postoperatively for at least 28 months (range 28–65 months). The demographics and pre- and postoperative data of the 36 patients are presented in Table 1.

**Table 1:** Summary of the demographics and surgery details

Variable	Details
No. of patients, *n* ^a^	36
Mean age at surgery (range), years^b^	48.64 ± 5.28 (39–60)
Sex (M/F, cases)^a^	21/15
Symptom^a^	
Myelopathy	19
Radiculopathy	14
Both	3
Levels^a^	
C3-C4, C5-C6 CDA; C4-C5 ACDF	8
C4-C5, C5-C6 CDA; C6-C7 ACDF	8
C4-C5, C6-C7 CDA; 5-C6 ACDF	16
C4-C5 CDA; C5-C6, C6-C7 ACDF	4
Mean operation time (range), min^b^	121.17 ± 16.74 (95–160)
Mean blood loss (range), ml^b^	299.58 ± 66.82 (210–500)
Mean follow-up (range), months^b^	41.11 ± 8.82 (28–65)
Hospital stay (range), days^b^	10.00 ± 2.70 (6–15)

*ACDF* anterior cervical discectomy and fusion, *CDA* cervical disc arthroplasty

^a^Data are displayed as a number

^b^Data are displayed as means ± standard deviation

The mean NDI and JOA scores for all cases improved significantly after surgery (*P* < 0.05) and were maintained at favorable levels within the follow-up period. The JOA score improvement rate was 70.83 % at the final follow-up examination. The data trends show a rapid decrease in the NDI scores and a rapid increase in the JOA scores immediately after surgery and a slow change in these scores during the follow-up period (Table 2).

**Table 2:** Pre- and postoperative NDI, JOA scores, and JOA scores IR (means ± standard deviation)

	Preoperative	Postoperative follow-up				
		1 month	3 months	6 months	12 months	Last follow-up
NDI (%)^a^	61.17 ± 3.54	33.61 ± 3.17	30.53 ± 2.91	23.86 ± 2.54	21.08 ± 2.30	18.64 ± 2.73
JOA^a^	9.39 ± 0.84	12.14 ± 1.36	12.44 ± 1.36	13.47 ± 1.08	14.19 ± 1.19	14.78 ± 1.27
JOA IR^b^		36.14	40.08	53.61	63.07	70.83

*NDI* Neck Disability Index, *JOA* Japanese Orthopaedic Association, *IR* improvement rate

^a^Data are displayed as means ± standard deviation

^b^Data are displayed as a percentage

Radiological evaluation was conducted by a senior spine surgeon who was not familiar with the patients’ situations to avoid information bias and reduce errors. An average ROM was calculated from three repeated measurements. The mean preoperative C2-C7 ROM of all cases was 46.39 ± 2.41°, and the postoperative mean values were 27.58 ± 5.82°, 31.78 ± 5.82°, 36.03 ± 4.93°, 46.03 ± 4.64°, and 47.50 ± 4.59° at the 1-, 3-, 6-, 12-month, and final follow-up examinations, respectively (Table 3). As shown in Table 3, cervical motion was significantly limited immediately after operation and was subsequently recovered to preoperative levels after 12 months (*P* = 0.721).

**Table 3:** Pre- and postoperative ROMs of C2-C7 and the superior and inferior adjacent segments

	Preoperative	Postoperative follow-up				
		1 month	3 months	6 months	12 months	Last follow-up
C2-C7 ROM (°C)	46.39 ± 2.41	27.58 ± 4.27*	31.78 ± 5.82*	36.03 ± 4.93*	46.03 ± 4.64	47.50 ± 4.59
SAS ROM (°C)	14.25 ± 1.81	6.56 ± 1.86*	9.67 ± 2.74*	14.03 ± 1.46	14.58 ± 1.34	15.00 ± 1.15
IAS ROM (°C)	10.89 ± 1.65	6.75 ± 1.70*	8.81 ± 2.16*	10.75 ± 2.37	11.06 ± 1.91	11.47 ± 1.84

Data are displayed as means ± standard deviation

*ROM* range of motion, *SAS* superior adjacent segment, *IAS* inferior adjacent segment

*Comparison between pre- and postoperative: *P* < 0.05

The ROMs of the superior and inferior adjacent segments were significantly decreased 1 month postoperation (*P* < 0.05). By contrast, the ROMs at 6, 12, and 24 months postoperation did not differ significantly from the preoperative ROMs, indicating that the ROMs returned to preoperative levels after 6 months (*P* > 0.05) (Table 3).

During the follow-up period, heterotopic ossification occurred in three patients without the need for further intervention. Symptomatic adjacent segment degeneration was encountered in two cases, and one of these required a second surgical treatment. Lateral radiographs showed a mild disc prosthesis migration (<3 mm) in two patients without obvious symptoms at the 6-month follow-up examination. No pseudarthrosis or other device migration was seen during the follow-up period. Vertebral stability and implant fusion were satisfactory in all cases at the last follow-up examination.

## Discussion

While ACDF continues to be the gold standard for the treatment of single- or double-level cervical spondylosis [4], it has been shown to alter spinal biomechanics, restrict intervertebral activity of the segment at the surgical level, and increase the rate of adjacent segment degeneration in a large number of cases [13–15]. Furthermore, several studies [16, 17] have indicated that challenging fusion and pseudarthrosis are more likely to occur when multiple segments undergo operative fusion, especially for three or four levels. Hence, there is a need for alternative surgical methods that can preserve segmental flexibility of the operative levels and reduce adjacent segment degeneration. Many researchers have reported that CDA is an effective alternative procedure that achieves equivalent or superior clinical and radiographic results compared to ACDF [9, 18]. Coric et al. [19] reported that CDA-treated patients (136 cases) had a lower incidence of adjacent segment degeneration compared to ACDF-treated patients (133 cases) in a randomized controlled trial with a minimum 2-year follow-up. Unfortunately, higher surgical requirements and increased prosthesis-related complications precluded its use in patients with multi-level CDA.

Currently, there is debate over the optimal surgical protocol for the treatment of cervical spondylosis involving three or more levels. CDA coupled with ACDF (hybrid surgery) considerably reduced the incidence of complications from multi-level fusion and largely preserved the physiological curvature of the cervical spine [8]. Sasso et al. [20] suggested that hybrid surgery not only maintains cervical activity after ACDF and avoids segment degeneration but also makes up for a lack of use of CDA. One study [9] indicated that hybrid surgery was comparable to ACDF and CDA in terms of safety and feasibility after conducting follow-up patient evaluations for a minimum of 2 years.

In the present study, 36 patients showed obvious improvements in postoperative NDI and JOA scores compared to preoperative scores. JOA score IRs were particularly high at the last follow-up evaluation (70.83 %). Neurological recovery during the follow-up period varied among patients but was satisfactory overall. The outcomes of this current study are very similar to those of a study by Shin et al. [21], who reported that hybrid surgery involving two segments resulted in a favorable recovery of NDI scores and eased neck and shoulder pain. Accordingly, we may reasonably conclude that the hybrid constructs offered favorable nerve root decompression and relieved neural symptoms.

Radiographic examinations showed that surgical segments were stabilized postoperatively and that the height of the intervertebral space of the replacement segments was basically consistent with the adjacent segments. Moreover, cervical spine motor function, C2-C7 ROM, and ROM of the superior and inferior adjacent segments were maintained at acceptable levels. The mean C2-C7 ROM, which was 46.39 ± 2.41° before the operation, was recovered after 12 months (46.03 ± 4.64°) and was maintained at the last follow-up evaluation (47.50 ± 4.59°). We noted that the ROM of the superior and inferior adjacent segments, which was 14.25 ± 1.81° and 10.89 ± 1.65° before the operation, respectively, was recovered after 6 months (14.03 ± 1.46° and 10.75 ± 2.37°, respectively) and increased at the last follow-up evaluation (15.00 ± 1.15° and 11.47 ± 1.84°, respectively). Although disc prosthesis migration was observed in two patients (<3 mm), there were no corresponding clinical signs or symptoms. In our study, one patient required a secondary operation for adjacent segment degeneration, possibly caused by an increased load of adjacent segment intervertebral activity after two-cage fusion.

Based on previous research and surgeons’ clinical experience with ACDF and CDA, clinical indications and contraindications have been drafted for these treatments [8]. CDA is considered a reasonable option for patients with a simple herniated disc without significant joint instability or facet joint degeneration, in particular young and middle-aged patients (less than 60 years old). On the contrary, ACDF or ACCF is preferable when the degree of intervertebral joint activity is restricted to 3°, with or without joint degeneration, to avoid heterotopic ossification. Other contraindications for CDA include obvious degeneration in segments adjacent to the surgical level, disc calcification, extensive spinal stenosis with osteophyte formation in the posterior of the vertebral body, and ossification of the posterior longitudinal ligament.

In this study, patients demonstrated satisfactory recovery of neurological function and favorable imaging results. This study had a large sample size of 36 patients with cervical spondylosis involving three contiguous segments, who were treated with hybrid surgery with a minimum follow-up period of 28 months. The major limitations of this study were its retrospective nature and the lack of a control group. Therefore, future studies with long-term follow-up and a control group with ACDF or CDA are necessary.

## Conclusions

Hybrid constructs can achieve thorough decompression of lesion segments, preserve the activity of non-fusion segments, and reestablish spinal stability. These results indicate that hybrid reconstructive techniques seem to be a promising, acceptable, and alternative approach for the treatment of multi-level cervical disc disease.
